# Sleep Difficulties in Preschoolers with Psychiatric Diagnoses

**DOI:** 10.3390/ijerph16224485

**Published:** 2019-11-14

**Authors:** Gabrielle Chénier-Leduc, Marie-Julie Béliveau, Karine Dubois-Comtois, Bryan Butler, Claude Berthiaume, Marie-Hélène Pennestri

**Affiliations:** 1 Department of psychology, Université de Montréal, Pavillon Marie-Victorin, C. P. 6128, succursale Centre-ville, Montréal, QC H3C 3J7, Canada; gabrielle.chenier-leduc@umontreal.ca (G.C.-L.); marie-julie.beliveau@umontreal.ca (M.-J.B.); 2 Hôpital en santé mentale Rivière-des-Prairies, CIUSSS du Nord-de-l’Ile-de-Montréal, 7070 Boulevard Perras, Montréal, QC H1E 1A4, Canada; karine.Dubois-Comtois@uqtr.ca (K.D.-C.); bryan.butler@mail.mcgill.ca (B.B.); claude.berthiaume.hrdp@ssss.gouv.qc.ca (C.B.); 3 Department of Psychology, Université du Québec à Trois-Rivières, 3351 Boulevard des Forges, Trois-Rivières, QC G8Z 4M3, Canada; 4 Department of Educational and Counselling Psychology, McGill University, Education Building, Room 614, 3700 McTavish Street, Montréal, QC H3A 1Y2, Canada

**Keywords:** sleep problems, preschoolers, psychiatric disorders, child psychiatry

## Abstract

**Background:** Sleep problems among preschoolers are highly prevalent. Given the impact of poor sleep quality on development, this relationship is particularly relevant in vulnerable populations but is less documented. This study aims to document parental perception of sleep problems in preschoolers assessed in a psychiatric clinic, as a function of diagnosis type. **Methods:** Children (14–71 months, *n* = 228) were evaluated by a psychiatrist, and diagnoses were pooled into four categories: behavioral disorders, relational disorders/psychosocial problems, developmental coordination disorder (DCD), and communication disorders. Sleep problems were measured using the Child Behavior Checklist (CBCL). Results: In this clinical sample of preschoolers, 21.6% of children were identified as having a sleep problem by their parents. Behavioral disorders and communication disorders were associated with increased parental report of sleep problems (respectively, trouble falling asleep and nighttime awakenings), while DCD was associated with lower parental report of sleep problems (fewer nighttime awakenings and less difficulty falling asleep) (*p* < 0.05). Relational disorders were not associated with parental reports of sleep difficulties (*p* > 0.05). Moreover, some psychiatric categories were associated with specific sleep symptoms (such as difficulty falling asleep and night awakenings). **Conclusion**: Parents of preschoolers with behavioral disorders and communication disorders are more likely to report sleep problems in their children than parents of preschoolers with DCD and relational disorders. Since different categories of psychiatric disorders are associated with specific types of sleep complaints, screening, and treatment should be adapted accordingly.

## 1. Introduction

Studies have confirmed the importance of sleep in children by documenting the association between lack of sleep and poorer physical health [1]. Moreover, sleep plays a crucial role in the maintenance of optimal mental health. For instance, short sleep duration and sleep disturbances in school-aged children and adolescents are associated with increased emotional (e.g., increased depression and anxiety symptoms) and behavioral difficulties (e.g., oppositional and inattentive behavior) [2,3,4]. Furthermore, sleep disturbances have been linked to academic difficulties, dysfunctional cognitive processes [4,5], decreased performance of motor tasks [6], and impaired verbal fluency and creativity [7].

While studies have examined the association between sleep quality and psychological functioning in school-aged children and adolescents, few studies have investigated this association in cohorts of preschoolers. Sivertsen et al. [8] conducted an extensive population-based study assessing sleep patterns at 18 months in 32,662 Norwegian toddlers and subsequent psychological outcomes at five years of age, using parent-report measures. They showed that sleep problems at 18 months, particularly short sleep duration and night awakenings, predicted the onset of behavioral and emotional problems at five years of age [8]. 

In preschoolers, the prevalence of sleep difficulties is high, ranging from 10% to 40% [9,10,11,12,13]. Difficulties include bedtime behavioral problems, excessive daytime sleepiness, fragmented sleep (nighttime awakenings), sleep-schedule irregularity, short sleep duration, and primary behavioral insomnia [11,14]. In parallel, epidemiological studies based on community samples also show moderate prevalence rates of psychiatric disorders, ranging from 13 to 27% in this age group [15,16,17]. Despite the consequences of both sleep problems and psychiatric disorders in preschoolers, knowledge is limited regarding their association. The understanding of this association is crucial, as psychiatric disorders observed in preschoolers are expected to persist in time [18] and can be exacerbated by sleep problems [3].

A recent study showed that 41% of preschoolers admitted to an early childhood psychiatric day treatment program suffered from insomnia (*n* = 183, mean age = 50.4 months) [19]. However, this sample only included children with emotional and behavioral diagnoses and therefore excluded children with developmental coordination disorder and communication disorders. In a study documenting sleep quality and duration in a sample of 194 preschool children aged between 2.0 and 5.5 years old, authors compared subjective and objective measures of sleep between three groups: children with autism spectrum disorder (ASD), children with developmental delays (DD), and typically developing children [20]. Compared to typically developing children, participants in the ASD group had less total sleep time during a 24-h period, whereas children in the DD group were characterized by frequent and longer awakenings after sleep onset. These findings suggest that different psychiatric diagnostic categories such as emotional, behavioral, and developmental disorders are characterized by specific sleep-related symptoms. However, studies evaluating sleep problems in other categories of neurodevelopmental disorders, such as communication or coordination disorders, are lacking in preschool children. It is important to include these categories while studying sleep problems as communication and coordination disorders are common among children, with respective prevalence rates of 5–8 % (preschool-aged children) [21] and 5–6% (school-aged children) [22]. Collectively, these findings emphasize the importance of screening for sleep problems in pediatric psychiatric clinics. 

Screening measures, including sleep-related questions, are used routinely within these clinics, such as the Child Behavior Check List (CBCL). Although some authors have argued that parents may have a biased perception of sleep patterns in childhood [23], specific sleep items of the CBCL are associated with other validated sleep measures such as sleep diaries, actigraphy, and polysomnography in school-aged children [24]. Consequently, there is a need to evaluate the CBCL’s discriminating power with various clinical populations. While studies often compare clinical populations to control groups, clinical groups are rarely compared with each other. Lack of such comparisons does not allow for verification of whether findings are relevant for a specific clinical group or for clinical children in general. Comparisons between clinical groups are needed to understand the reality of the pediatric psychiatric population (considering the high comorbidity rate) and to identify specific sleep difficulties among subgroups of clinical children. Categories of psychiatric diagnoses from the Diagnostic and Statistical Manual of Mental Disorders have been used to study psychiatric symptoms in young children [17]. However, studies rarely include more than one diagnostic category at a time [25]. To our knowledge, no study has examined parental perception of sleep disorders in a population of young children belonging to four broad categories of psychiatric diagnoses: (1) behavioral disorders; (2) relational disorders and psychosocial problems; (3) developmental coordination disorder; (4) communication disorders.

The aims of the present study are: (1) to document the parental perception of sleep difficulties in preschoolers referred to an early childhood mental health outpatient clinic; (2) to determine if sleep difficulties vary according to the type of psychiatric diagnosis (e.g., behavioral disorders, relational disorders and psychosocial problems, developmental coordination disorder, communication disorders); (3) to examine if each type of psychiatric diagnostic category is associated with specific sleep disturbances (falling asleep, sleep quality, and parasomnia); (4) to assess the impact of cumulative psychiatric diagnostic categories on sleep difficulties. 

## 2. Materials and Methods

### 2.1. Participants 

Preschoolers from a large metropolitan area referred by their physician for psychiatric evaluation were sent to the early childhood psychiatric clinic. The Research Ethics Board of the Hôpital en Santé Mentale Rivière-des-Prairies authorized access to the clinical records of 296 patients between one and six years old, assessed between July 2006 and September 2009. Records with missing information (diagnosis, sleep, demographics) were excluded (*n* = 19). Children with an ASD diagnosis (*n* = 24), intellectual disability (*n* = 14), or with a diagnosis other than the four categories used in the present project (*n* = 11) were also excluded for a final *n* of 228 patients. Since lower socioeconomic status (SES) and family environment are known to be associated with more sleep-wake problems [26,27,28], mother’s level of education and family composition (biological family, other family composition) were also retrieved from the clinical records. 

### 2.2. Psychiatric Diagnosis

Following initial assessment, diagnoses were established by the child psychiatrists and subsequently organized into four categories defined by the DSM-IV-TR’s structure (valid system at the time): (1) behavioral disorders (oppositional defiant disorder, disruptive behavior disorder not otherwise specified); (2) relational disorders and psychosocial problems (e.g., parent-child relational problems, neglectful parental conduct/educational problems or severe psychosocial problems); (3) developmental coordination disorder (DCD); (4) communication disorders (mixed receptive-expressive language disorder, expressive language disorder, phonological disorder). The two psychiatrists cumulated more than 40 years of experience and were always assisted by another health professional specialized in mental health (i.e., a nurse). To assess the impact of multiple psychiatric diagnoses, children were assigned a score of 1 to 4 according to the number of categories in which they had a positive diagnosis. Therefore, children with two diagnoses within the same category were attributed a score of 1. 

### 2.3. Sleep

The Child Behavior Checklist (CBCL) 1.5/5 years was systematically sent to parents before the first appointment. This widespread, validated, and standardized 100-item parent-report measure of behavioral, emotional, and social problems in young children includes a sleep problems subscale. This subscale includes seven items related to different aspects of sleep: Doesn’t want to sleep alone, Has trouble falling asleep, Has nightmares, Resists going to bed at night, Sleeps less than others, Talks or cries in sleep, and Wakes often at night. Parents assessed the presence and severity of the child’s symptoms on a Likert scale of “0 = Not True, 1 = Somewhat or Sometimes true, and 2 = Very true or Often true.” A total score of ≤7 refers to the subclinical range, a total score of 8 represents the borderline range, and the clinical range is reached when the total score is ≥9. In the present study, a threshold of ≥8 (including both borderline and clinical range) was used, defined as problems that are a source of concern according to The Achenbach System of Empirically Based Assessment (ASEBA) manual for the CBCL 1.5/5 years [29]. The sleep problems subscale’s test-retest reliability is 0.92, cross-informant agreement is 0.59, and stability is 0.60 over a one-year period [30]. Raw scores were used in statistical analyses to preserve the full range of variation [31].

### 2.4. Data Analyses

To test whether socio-demographic characteristics of the sample were related to sleep problems, correlations and independant *t*-tests were first undertaken for child age and sex, family type, and mother’s level of education. To document the overall proportion of sleep difficulties, the percentage of children presenting with parental-reported sleep problems was first calculated using the whole sample. Next, to determine if sleep difficulties were associated with specific categories of psychiatric diagnoses, a linear regression analysis was conducted on the continuous score of the sleep problems subscale by entering all four categories of psychiatric diagnoses in the same model. This analytic strategy allows controlling for shared variance between diagnostic categories. To further document the association between each CBCL sleep item and psychiatric category, multinomial logistic regression analyses were performed separately on each specific sleep item using the four diagnostic categories as predictors. Results include two odds ratios for each of the four predictors using the following comparisons: (1) Sometimes versus Never and (2) Often versus Never. The impact of belonging to multiple diagnostic categories on sleep difficulties was assessed using a one-way ANOVA, with the number of clinical categories as the independent variable. Statistical analyses were conducted using SPSS statistics 25.0 (IBM Corp., Armonk, NY, USA). 

## 3. Results

### 3.1. Descriptive Data

The final sample consisted of 228 participants (14 to 71 months; Mean = 47.79 months, SD = 13.62; 169 boys). More than half of mothers possessed post-secondary education (Table 1). Most children were living with their biological parents (Table 1). In our final sample, 39.0% of children were diagnosed with a behavioral disorder, and 44.7% received a diagnosis within the relational problems category. Developmental coordination disorder and communication disorders were highly prevalent, with 76.8% and 77.6% of children receiving a positive diagnosis in these categories, respectively. Comorbidity was high, with 89.9% of children receiving a diagnosis in at least two different categories. Results of t-tests and correlations revealed no significant association between sleep problems and child sex (T(226) = 0.769, *p* = 0.44) or age (*r =* −0.018, *p =* 0.78). Family type (F(2, 223) = 3.39, *p* = 0.04) and mothers’ level of education (r = −0.15, *p* = 0.03) were significantly related to sleep problems and were therefore included in further analyses.

### 3.2. The Proportion of Sleep Problems in Preschoolers with Psychiatric Disorders and the Association between Parental-Reported Sleep Problems and Diagnostic Categories

One out of five children in our sample reached either the borderline (5.7%) or clinical (15.9%) threshold for sleep problems (for a total of 21.6%), as reported by parents. According to the regression analysis model, the association between diagnostic categories and sleep problems (while controlling for family type and mother’s level of education) was significant, R squared change = 0.077, F(4, 197) = 4.30, *p* < 0.01. Table 2 shows that having a diagnosis in the behavioral disorder (*p* < 0.05) and the communication disorder (*p* < 0.05) categories significantly predicted the presence of parental reported sleep problems. However, having a diagnosis of DCD was associated with fewer parental reports of sleep problems (*p* < 0.01). Relational disorders were not associated with parental reports of sleep problems (*p* > 0.05).

### 3.3. Association between Specific Sleep Problems and Diagnostic Categories

Results of the multinomial logistic regression analyses showed that the overall model was significant for three of the seven items, namely Has trouble falling asleep (X^2^_(12)_ = 29.07, *p* < 0.01), Wakes often at night (X^2^_(12)_ = 23.05, *p* < 0.05) and Talks or cries in sleep (X^2^_(12)_ = 25.65, *p* < 0.01). The models predicting other sleep problems (i.e., Doesn’t want to sleep alone, Resists going to bed at night, Has nightmares, Sleeps less than others) were not significant (X^2^_(12)_ between 12.63 and 17.48; *p* > 0.05). 

As shown in Table 3, further analyses revealed that DCD and behavioral disorders were significant predictors of having Trouble falling asleep. Parents of children with DCD were about five times less likely to report *Often* versus *Never* for the item Trouble falling asleep (OR = 0.19, 95% CI: 0.05–0.71). Parents of children with behavioral disorders had an increased chance of endorsing *Often* (OR = 2.18, 95% CI: 1.01–4.70) and Sometimes (OR = 2.21, 95% CI: 1.09–4.49) versus *Never* for the item Trouble falling asleep. The OR of parents reporting Wakes often at night as opposed to *Never* was 6.80 in communication disorders (95% CI: 1.39–33.36). Parents of children with DCD had about 6.5 times less chance of endorsing *Often* versus never (OR = 0.15 95% CI: 0.04–0.62) for the item Wakes often at night. Concerning the item Talking or crying during sleep, despite an overall significant model, further analysis revealed no significant predictors (see Table 3). 

### 3.4. Effect of Cumulating Positive Diagnostic Categories on Sleep Difficulties

**Figure 1:**
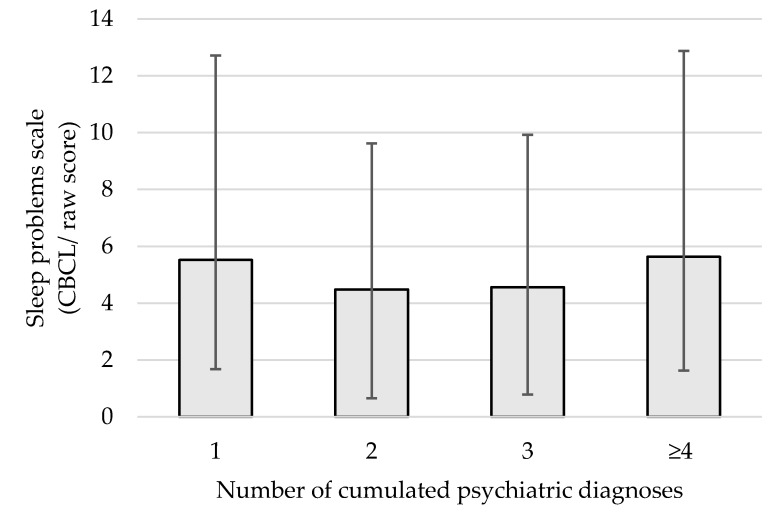
Mean total raw scores on the Child Behavior Checklist (CBCL) sleep problems scale according to the number of cumulated psychiatric diagnostic categories.

The mean raw total score on the CBCL sleep problems scale did not vary as a function of the number of cumulated psychiatric diagnostic categories, F(3, 198) = 0.683, *p* = 0.56 (Figure 1).

## 4. Discussion

### 4.1. The Proportion of Sleep Problems in Preschoolers with Psychiatric Disorders

The proportion of sleep disorders based on the CBCL in our sample of preschoolers referred to an early childhood mental health clinic was 21.6%, including both the borderline (5.7%) and clinical range (15.9%). This proportion is similar to the prevalence found throughout childhood in community samples, which range from 10 to 25% [9,10]. As hypothesized, characteristics of sleep problems varied according to a diagnostic category, suggesting that interventions could be tailored for specific psychiatric diagnoses.

### 4.2. Sleep Problems and Behavioral Disorders

The presence of behavioral disorders in children predicted an increase in parental reports of sleep problems. Oppositional defiant disorder (ODD) is the main diagnosis composing this category. The present results are consistent with studies showing that ODD is associated with significantly higher levels of concurrent sleep problems in older children aged 9 to 16 years [32]. Sleep problems were also associated with behavioral problems in a community sample of preschoolers aged four to five years [33]. The authors of this last study hypothesized that an irregular sleep schedule might contribute to the association between sleep problems and behavioral adjustment. Interventions addressing sleep schedules in children with a diagnosis of DCD could be explored.

More specifically, parents of children with behavioral disorders were more likely to report that their child has Trouble falling asleep. A recent study conducted on adolescents also showed that ODD was specifically associated with difficulty falling asleep and restless sleep [32]. Another study highlighted the association between behavioral problems and sleep problems in children aged seven to 12 diagnosed with conduct disorder and oppositional defiant disorder. Children with shorter sleep duration were more aggressive and showed more rule-breaking behavior than children with more sleep, as reported by their parents [34]. The present results support these associations between sleep problems and ODD in preschoolers. Some authors have suggested that oppositional children are more likely to resist going to bed and that the implementation of a more regular sleep schedule increases the manageability of their behavior [33]. Since sleep problems tend to persist over time [10], the present results suggest that sleep initiation difficulties should be targeted as early as possible in preschoolers with behavioral disorders.

### 4.3. Sleep Problems and Relational Disorders and Psychosocial Problems

Relational disorders and psychosocial problems are described as “a situation in which emotionally attached individuals engage in communication or behavior patterns that are destructive or unsatisfying” [35]. In the current sample, receiving a diagnosis in the relational disorders or psychosocial problems category was not associated with parental reports of sleep difficulties. Other studies have shown an association between sleep quality and parent-child relationships [36,37], and a recent review paper highlighted the impact of behavioral sleep interventions for bedtime problems and night awakenings on secondary outcome variables such as parent-child relationships [13]. Differences in the population and type of measure (sleep and relational variables) could potentially explain the presence or absence of associations between relational disorders and sleep problems.

### 4.4. Sleep Problems and Developmental Coordination Disorder

Contrary to other diagnostic categories, parental reports of children with developmental coordination disorder (DCD) were associated with fewer sleep difficulties. The items, Wakes often at night and Trouble falling asleep, were endorsed less frequently. However, when compared to non-clinical samples, children with developmental coordination disorders often show bedtime resistance, more parasomnias, and increased daytime sleepiness [38]. Furthermore, parents of older children with DCD (seven to 16 years old) reported more sleep disturbances in their child than parents of typically developing children [39]. Another study conducted in children aged eight to 12 years also showed higher sleep disturbance scores in children with DCD compared to a control group [38]. The different age range used could potentially explain these conflicting results. Alternatively, motor difficulties may contribute to children being more tired in the evening and therefore falling asleep more easily at night. These discrepancies could also result from our sample being composed exclusively of children with a psychiatric diagnosis, as opposed to a comparison between a clinical sample and typically developing peers. The present results shed new light on sleep difficulties of preschool children aged 14 to 71 months with various psychiatric diagnoses compared to previously published data on older children.

### 4.5. Sleep Problems and Communication Disorders

Having a positive diagnosis in the communication disorder category was associated with higher overall parental reports of sleep problems and was a significant predictor of the individual item Wakes often at night. When compared to typically developing peers, children aged 24 to 26 months with expressive language delay had a higher total score on the CBCL sleep problems scale [39]. Other studies have also shown an association between sleep and language ability. Sleep disturbances in children have been linked to impaired semantics, syntax, phonology, morphology, and pragmatics [40,41,42]. In a recent study, older children (three to 18 years) with developmental communication disorder presented with more sleep problems than children in the control group, specifically trouble to fall asleep and waking up earlier [42]. Again, the control group in this study was composed of typically developing children, as opposed to a clinical population, as in the present study. However, a small sample size (*n* = 28), comorbidity with ASD (*n* = 8), and the fact that clinical status was solely determined by parental report limit their conclusions. These results highlight the importance of comparing clinical groups while considering comorbidities and stress the importance of targeting the continuity of sleep in children with communication disorders.

### 4.6. Effect of Cumulating Positive Diagnostic Categories on Sleep Difficulties

The present results showed no effect of cumulating multiple diagnostic categories on parental reports of sleep problems. Therefore, parents of young children who have a single positive diagnosis will report sleep problems to the same degree as parents of children having two or more diagnoses in different categories. This finding emphasizes the importance of assessing sleep quality, even in the presence of only one psychiatric disorder.

### 4.7. Limitations and Future Research

While measuring sleep with the CBCL might raise concerns, it is a time and cost-effective method of assessing the parental perception of children’s sleep patterns. Moreover, items on the CBCL provide specific information about various types of sleep complaints. Therefore, this measure is a helpful tool in clinical, epidemiological, or archival studies when objective methods are not available [43].

Boys were over-represented in our sample, which is consistent with other clinical samples [44,45]. It is also representative of the sex differences which exist in the prevalence of many diagnoses included in the psychiatric categories. For example, DCD is known to be more prevalent in boys [46]. Comorbidity rates are high in our sample, which is consistent with the fact that, in a clinical sample, boys are more likely to have multiple diagnoses [44]. Also, the high proportion of developmental disorders (such as communication and motor coordination disorders) in our sample is consistent with results obtained in clinically referred children [45].

One limitation of the present study is the lack of a control group, which would have enabled us to compare the prevalence rate of sleep problems between children with psychiatric diagnoses and typically developing children while also allowing for comparison between clinical groups. Furthermore, a longitudinal study design would have allowed for a better understanding of how sleep problems and psychiatric disorders influence each other. Other variables of interest, such as maternal age and birth weight, could also enrich future studies.

## 5. Conclusions

As is the case in healthy samples, sleep problems are highly prevalent in preschoolers diagnosed with psychiatric disorders. A better understanding of the relationship between sleep and psychiatric disorders in this vulnerable population will provide insight into the use of more specific and appropriate interventions. This study should be replicated with a combination of subjective and objective sleep measures in association with specific diagnoses. Finally, it is imperative to investigate qualitative differences in sleep problems that vary across psychiatric diagnoses to tailor specific interventions.

## Figures and Tables

**Table 1:** Demographic and socioeconomic variables.

Age (months) *N* %	*N*	%
<18 months	4	1.8
18 to <24	5	2.2
24 to <36	32	14.0
36 to <48	70	30.7
48 to <60	65	28.5
60 to <72	52	22.8
**Sex**		
Boys	169	74.1
Girls	59	25.9
**Number of Categories with Positive Diagnoses**		
1	23	10.1
2	119	52.2
3	62	27.2
4	24	10.5
**Post-Secondary Education**		
Mothers	127	55.7
**Family Composition**		
Biological family	155	68.6

**Table 2:** Association between diagnostic categories and sleep problems.

Diagnostic Categories	B	95% CI	Beta	*p*-Value
Behavioral disorders	1.08	(0.13, 2.04)	0.15	0.04
Relational disorders	0.62	(−0.48, 1.71)	0.09	0.27
Developmental coordination disorder (DCD)	−2.35	(−4.01, −0.69)	−0.29	0.01
Communication disorders	1.74	(0.02, 3.46)	0.21	0.05

**Table 3:** Association between the Child Behavior Checklist (CBCL) sleep problem syndrome scale items according to the diagnostic categories.

Diagnostic Categories	Has Trouble Falling Asleep		Wakes Often at Night		Talks or Cries in Sleep	
	X^2^_(2)_	*p*	X^2^_(2)_	*p*	X^2^_(2)_	*p*
Behavioral disorders	6.48	0.04	1.46	0.48	5.13	0.08
Relational disorders	1.16	0.56	0.20	0.90	3.39	0.18
Developmental coordination disorder (DCD)	8.76	0.01	7.65	0.02	3.92	0.14
Communication disorders	2.33	0.31	6.36	0.04	2.80	0.25
